# BRIDGE – Behavioral and physical activation for multimorbid older adults with depressive symptoms during the inpatient to outpatient transition: Study protocol for a multicenter two-arm randomized controlled trial

**DOI:** 10.1186/s12877-025-06949-8

**Published:** 2026-03-05

**Authors:** Alexandra Wuttke, Nils Henrik Pixa, Claudia Voelcker-Rehage, Lisa Marie Warner, Valentina Ludwig, Christine Müller, Brigitte Anderl-Doliwa, Andreas Fellgiebel, Peter Wagner, Jochen Heckmann, Marcus Unger, Ulrich Seidl, Sebastian Walther, Juergen Deckert, Alexandra Sibylle Herr, Jan Weyerhäuser, Felix Muehlensiepen, Kirsten Haas, Anna Schäfer, Peter Heuschmann, Julia Katharina Wolff, Lisa Voigt, Katharina Geschke, Eva-Marie Kessler

**Affiliations:** 1 Department of Psychology, University of Konstanz, P.O. Box 905, Konstanz, 78464 Germany; 2 Central Research Unit for Mental Health in Older Age, Department of Psychiatry and Psychotherapy, University Medical Center Mainz, Mainz, Germany; 3 Department of Psychology, MSB Medical School Berlin, Berlin, Germany; 4 Department of Neuromotor Behavior and Exercise, University of Münster, Institute of Sport and Exercise Sciences, Münster, Germany; 5 German Depression League (Deutsche DepressionsLiga e.V.), Bonn, Germany; 6 Pfalzklinikum, Service Provider for Mental Health & Neurology, Klingenmünster, Germany; 7 Agaplesion Elisabethenstift, Clinic for Psychiatry, Psychosomatics and Psychotherapy, Darmstadt, Germany; 8 Agaplesion Markus Krankenhaus, Clinic for Psychiatry, Psychosomatics and Psychotherapy, Frankfurt, Germany; 9 Landeskrankenhaus, Geriatric Specialist Clinic Rheinhessen-Nahe – Center for Acute Care and Rehabilitation, Bad Kreuznach, Germany; 10 SHG-Kliniken Sonnenberg, Saarbrücken, Germany; 11 Department of Psychiatry, Psychosomatics and Psychotherapy, University Hospital Würzburg, Center for Mental Health, Würzburg, Germany; 12 Landeskrankenhaus, Gerontopsychiatry Rheinhessen-Fachklinik Alzey, Alzey, Germany; 13 Center for Health Services Research, Faculty of Health Sciences Brandenburg, Brandenburg Medical School, Rüdersdorf, Brandenburg, Germany; 14 Department of Cardiology, Angiology and Intensive Care Medicine, Deutsches Herzzentrum der Charité, Berlin, Germany; 15 Julius-Maximilians-Universität Würzburg, Institute of Clinical Epidemiology and Biometry, Würzburg, Germany; 16 University Hospital Würzburg, Institute of Medical Data Science, Würzburg, Germany; 17 IGES Institute, Berlin, Germany; 18 Department of Prevention Research and Social Medicine, Institute for Community Medicine, University Medicine Greifswald, Greifswald, Germany

**Keywords:** Ageing, Behavioral change, Blended care, E-health, Mental health, Interdisciplinary, Patient involvement, Physical exercise, Prevention

## Abstract

**Background:**

Older adults with multimorbidity and depressive symptoms are particularly vulnerable during the transition from (partial) inpatient to outpatient care, with a high risk of readmission and rehospitalization due to substantial barriers in accessing appropriate mental healthcare. The BRIDGE intervention addresses this critical transition through a structured, manualized, interdisciplinary treatment program aimed primarily at improving depressive symptoms.

**Methods:**

BRIDGE is a 12-week blended care intervention that combines two evidence-based therapeutic strategies—behavioral activation (BA) and physical exercise (PE)—to promote structured daily activity and improve mood and physical health. The program is delivered by an interprofessional team consisting of a nurse, psychologist, and exercise scientist at each study center, and includes both home visits and digital components via a tablet-based e-health platform. Core elements comprise BA exercises in line with behavioral change techniques, and a video-guided home-based PE program. The multicenter two-arm randomized controlled trial evaluates the efficacy, feasibility, and cost-effectiveness of the BRIDGE intervention compared to treatment as usual. The post-discharge maintenance or improvement of the outcome of inpatient treatment for depressive symptoms is considered as the primary outcome. Secondary outcomes include reductions in recurrent hospital admissions, improvements in physical, emotional, and cognitive functioning as well as quality of life, and evidence of a favorable cost-effectiveness ratio.

**Discussion:**

BRIDGE offers a comprehensive, scalable intervention model that integrates behavioral and physical activation into the daily life of older patients with multimorbidity and depressive symptoms. By embedding hybrid delivery formats, structured interprofessional collaboration, and a focus on sustainable home-based routines, the intervention addresses the complex needs of older multimorbid adults during a critical care transition. Findings from this trial will inform future implementation in diverse healthcare settings and contribute to evidence-based service development in geriatric mental health.

**Trial registration:**

German Clinical Trials Register (DRKS-ID: DRKS00035625, Date of registration in DRKS: 2024-12-19, https://drks.de/search/en/trial/DRKS00035625/details).

**Supplementary Information:**

The online version contains supplementary material available at 10.1186/s12877-025-06949-8.

## Background

### Treatment gap in patients with late-life depression

Depression in older adults represents a critical public health issue, with the estimated average prevalence of late-life depression lying at 31.74% according to a recent global meta-analysis [1]. Notably, prevalence rates vary significantly depending on the care setting: For hospital settings, studies have reported prevalence rates of depressive symptoms at around 27% in geriatric patients [2] and up to 34% in psychogeriatric patients [3]. Independent of the setting, late-life depression has devastating consequences for older adults, including an increased risk of suicide and reduced physical, cognitive, and social functioning, all of which are in turn associated with higher service utilization, premature institutionalization, and mortality [4]. Patients with multimorbidity represent a particularly vulnerable population for the development of late-life depression [5], showing a two to three times higher risk of depression compared to individuals without multimorbidity [5]. At the same time, multimorbidity is associated with higher hospitalization and readmission rates [6]. Thus, a vicious cycle of multimorbidity, depression, and hospitalization is at play, underlining the need to target depressive symptoms in older adults in as timely a manner as possible.

However, late-life depression remains significantly underdiagnosed and inadequately treated across all care settings and patient populations [7]. Moreover, untreated depression in this population is associated with adverse health outcomes [8] — even at subclinical levels [9].

Treatment options for late-life depression include pharmacological and non-pharmacological interventions. However [8–11], home-dwelling older adults with multimorbidity and depressive symptoms face substantial barriers to accessing appropriate mental healthcare [12]. Particularly in view of the high rehospitalization rates of patients with multimorbidity [5], the transition from an inpatient to an outpatient care setting represents an especially vulnerable and often challenging period, marked by a lack of specialized follow-up services to consolidate treatment gains and to prevent relapse and symptom progression. During this transition, the treatment gap for older adults with depressive symptoms increases further. Evidence-based non-pharmacological approaches for treating depressive symptoms in older adults, such as behavioral activation (BA) and physical exercise (PE), do exist; however, they are often not adapted to the specific needs of multimorbid older individuals within the healthcare system.

### Psychosocial and behavioral interventions for late-life depression: behavioral activation (BA) and physical exercise (PE)

BA has been shown to be an effective intervention for treating depression in older adults. For instance, a systematic review and meta-analysis indicated that BA significantly reduces depressive symptoms among older individuals living in the community, with a standardized mean difference of −0.72 [8] and a randomized controlled trial (RCT) revealed that BA significantly improves mental health outcomes, including depression and stress, among older adults with subthreshold depression [13]. Moreover, a study focusing on older adults with type 2 diabetes and comorbid depressive symptoms found that BA improved subjective well-being and reduced depressive symptoms [14]. A meta-analysis of RCTs of BA treatments for depression highlighted that BA is a well-established alternative to cognitive therapy, with a pooled effect size indicating significant reductions in depressive symptoms [15]. Collectively, these findings underscore the potential of BA as a viable treatment option for older adults experiencing depression.

PE has likewise been recognized as an effective intervention for alleviating depressive symptoms with effects comparable to psychotherapy and pharmacotherapy as indicated by a recent meta-analysis [16]. Moreover, a systematic review showed that various forms of PE led to significant and clinically relevant improvements in depression among older individuals, with a pooled effect size of 0.52 [17]. Specifically, multicomponent exercise (e.g., the combination of aerobic, strength, and coordination exercise) and mind-body exercises (e.g., tai chi) were highlighted as particularly beneficial, yielding significant reductions in depressive symptoms [17, 18]. Furthermore, a systematic review indicated that physical activity interventions were effective in reducing depressive symptoms in the short term [19], while the long-term effects still require further investigation.

Overall, the evidence supports the use of PE as a promising treatment for depression in older adults, highlighting its potential benefits in improving mental health and overall well-being.

### The need for person-centered treatment options

Despite the effectiveness of these non-pharmacological interventions, the majority of research has focused on younger or middle-aged adults, often excluding older adults in general and those with significant comorbid conditions in particular [20]. Thus, older adults with multimorbidity are especially underrepresented in clinical trials, despite their high prevalence of depression and functional limitations [21, 22], highlighting the critical need for research that specifically targets this vulnerable population. The transition from inpatient to outpatient care marks an especially vulnerable phase, with a high risk of readmission and rehospitalization due to fragmentation of care across sectors [23, 24] especially for older adults [25]. Therefore, blended care models – combining in-person consultation and digital elements might provide low-barrier access for multimorbid patients during this transition [26, 27].

**Fig. 1:**
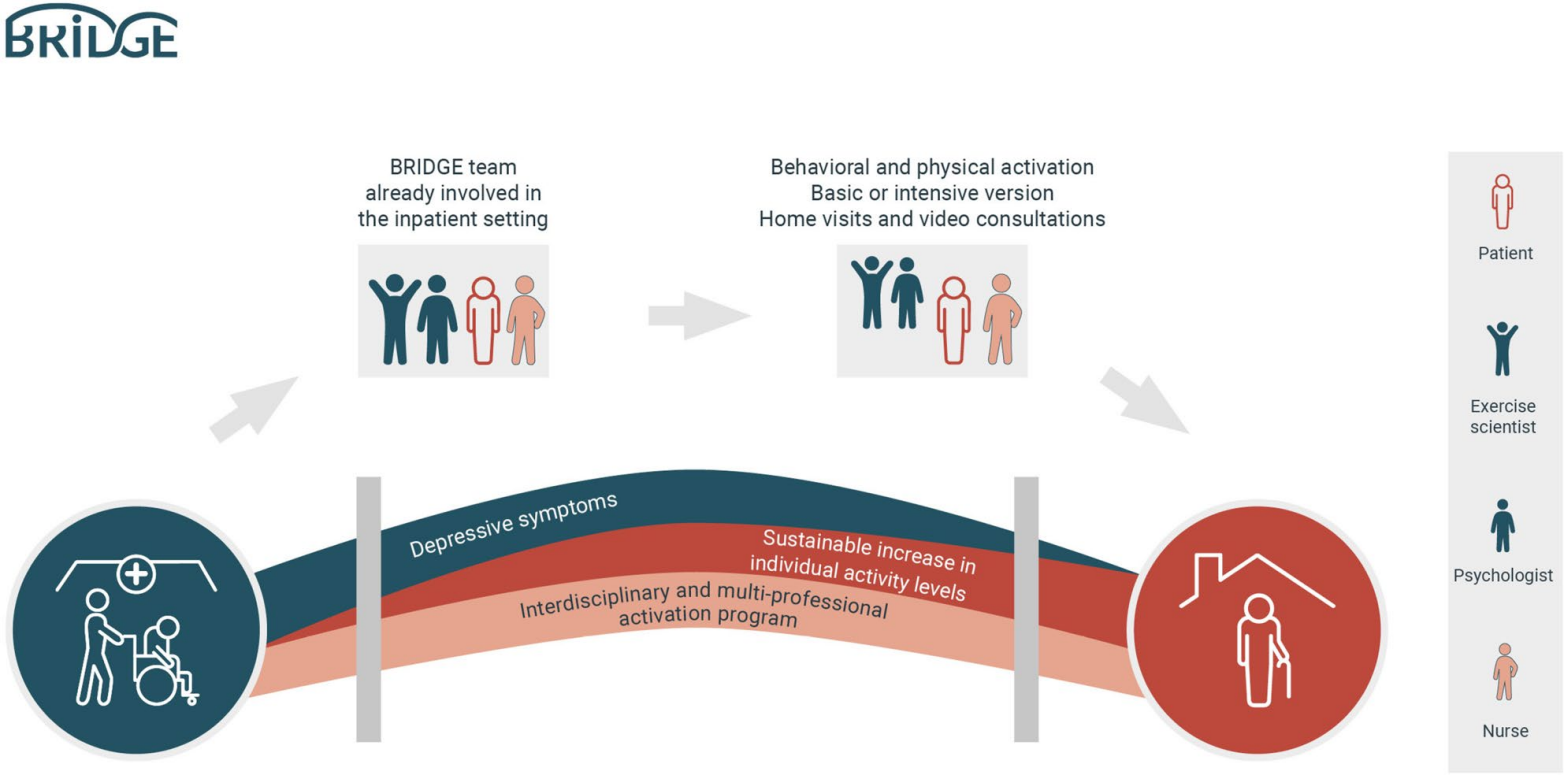
BRIDGE between hospital and home: The BRIDGE intervention links inpatient and home-based blended care through a multi-professional behavioral and physical activation program aiming to sustainably increase activity levels and reduce depressive symptoms in older multimorbid adults

BRIDGE was therefore developed to address this treatment gap for older adults with multimorbidity and depressive symptoms at the inpatient to outpatient transition (Fig. 1). At its core, it consists of an interdisciplinary team of a nurse, an exercise scientist, and a psychologist who deliver blended care, via home visits and video consultation, a three-month, home-based intervention consisting of an activation program including the combination of BA [21] and PE [19] in a synergistic application [28, 29].

### Objectives

The primary objective of this multicenter two-arm RCT is to evaluate the effectiveness – that is, assessing how well the three-month BRIDGE intervention performs under real-world conditions among a heterogeneous population of multimorbid older adults with depressive symptoms in maintaining or improving the results of inpatient treatment with regard to depressive symptoms. Secondary outcomes include reductions in recurrent hospital admissions, improvements in physical, emotional, and cognitive functioning as well as quality of life, and evidence of a favorable cost-effectiveness ratio.

### Primary hypothesis

Outcomes of inpatient treatment for depressive symptoms will be more likely to be maintained or to improve further in the intervention group (IG) than in the control group (CG), as measured from post-discharge up to six months post-discharge (see supplementary material for an overview of all secondary hypotheses).

## Methods

The study is funded by the German Innovation Fund of the Joint Federal Committee (Innovationsfonds des Gemeinsamen Bundesausschusses (G-BA) grant number: 01NVF23102) and registered in the German Clinical Trials Register (DRKS-ID: DRKS00035625, Date of registration in DRKS: 2024-12-19, https://drks.de/search/en/trial/DRKS00035625/details). It has received ethical approval from the following Ethics Committees of the appropriate State Chamber of Physicians in Rhineland-Palatinate (Landesärztekammer Rheinland-Pfalz, reference number: (2024–17741), in Hesse (Landesärztekammer Hessen reference number: 2024–3904), and Saarland (Landesärztekammer Saarland reference number: 171/24) in addition to the local Ethics Committees of the University Hospital of the University of Würzburg (reference number: 2024 − 233), and the MSB Medical School Berlin (reference number: 2024 − 211). This study protocol is reported in accordance with the current SPIRIT (Standard Protocol Items: Recommendations for Interventional Trials; 2025 [30]) guidelines and oriented on the CONSORT (Consolidated Standards for Reporting Trials; [31]) statement for transparent reporting of parallel-group randomized trials.

### Patient and public involvement

The BRIDGE intervention was developed and consolidated in close collaboration with the German Depression League (Deutsche Depressionsliga e.V.). As part of the preparatory phase, qualitative interviews were conducted with older adults with lived experience of depression. Furthermore, stakeholders and relevant professional groups (e.g., physicians, psychotherapists, exercise scientists, nurses) at the participating study centers were interviewed prior to finalizing the study protocol. Thus, BRIDGE was developed in an iterative manner with perspectives from patients, healthcare professionals, and health insurance companies.

### Study design

**Fig. 2:**
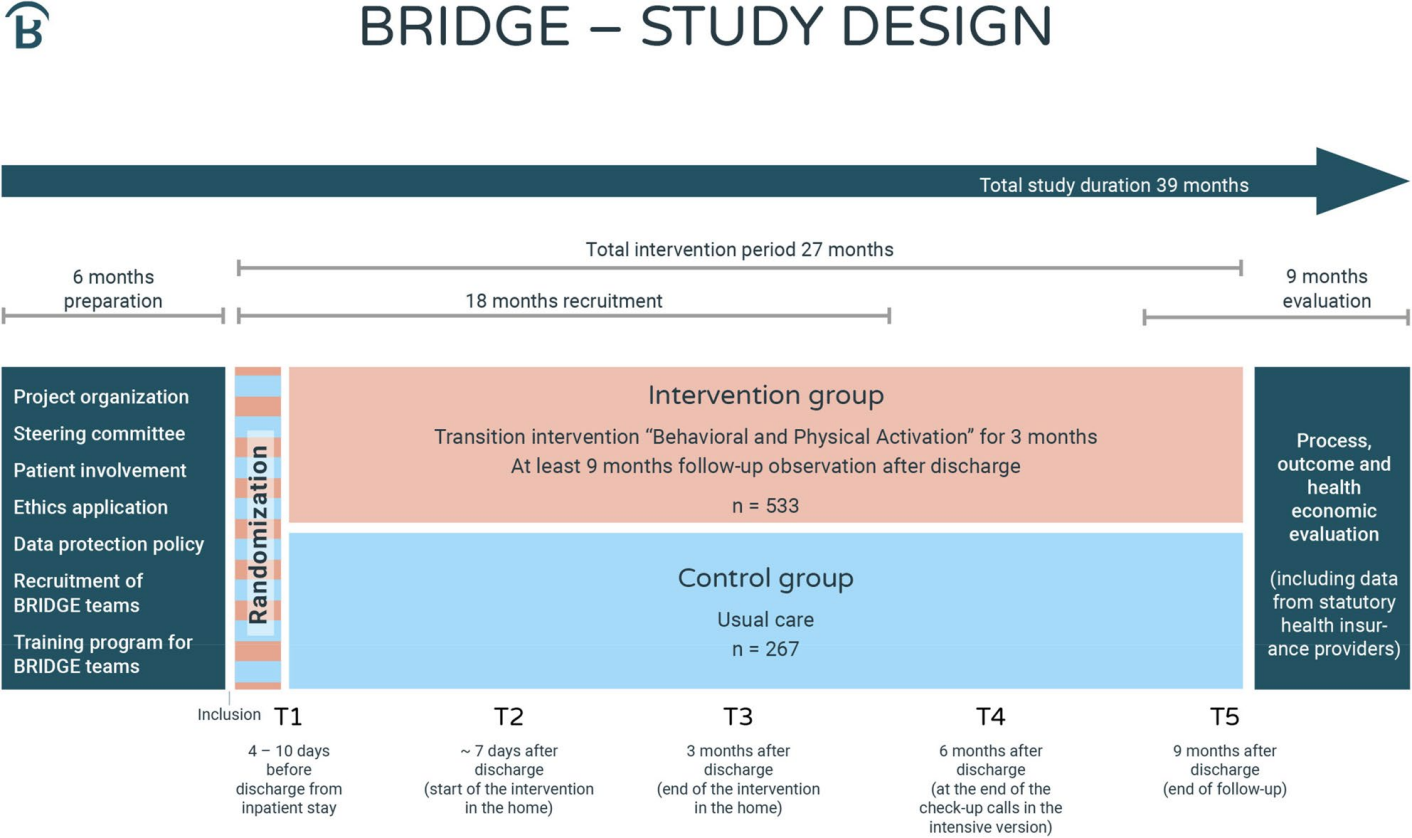
Overview of the BRIDGE study design with a total study duration of 39 months, including a 6-month preparation phase before an 18-month recruitment period, and a 27-month total intervention period. Participants are randomized to either the intervention or control group. Outcome assessments are performed before (T1, T2) and after (T3) the intervention or control condition, and at 6 (T4) and 9 (T5) months after discharge from (partial) inpatient care. The total intervention period is followed by a 9-months evaluation phase

The study is designed as a multicenter two-arm RCT with a total duration of 39 months including a 27-month intervention period (see Fig. 2). Within an 18-month recruitment period, participants will be recruited during their (partial) inpatient stay. That means participants can either be full inpatients or partial inpatients, i.e. attending a day clinic but returning to their home at night. Recruitment takes places at the seven clinical study centers across Germany that cover both urban and rural regions in the Federal states of Bavaria, Rhineland-Palatine, Hesse, and Saarland.

### Sample size calculation

Recent meta-analyses evaluating the combined effects of behavioral therapy and physical exercise on depressive symptoms [28, 32] reported a significant overall effect size of Hedges’ g = − 0.47 (95% CI: − 0.83 to − 0.12, *p* = 0.005). This corresponds to a moderate reduction in depressive symptoms in favor of the combined intervention. However, to account for the lack of evidence in older, multimorbid adults, the more conservative power analysis was based on detecting a small to moderate effect size (Cohen’s d = 0.3; corresponding odds ratio ≈ 1.7) for the primary outcome of maintenance or improvement of inpatient treatment results with respect to depressive symptoms (binary: success vs. no success). Hence, the a priori sample size calculation resulted in a total of 800 participants accounting for an anticipated dropout rate of 20% and a statistical power of 90%. 533 participants will be allocated to the IG and 267 to the CG, following a 2:1 randomization ratio. This ratio was chosen to conduct a subgroup analysis within the IG comparing a basic version to an intensive version of the intervention.

### Recruitment strategy

Participants will be recruited during their (partial) inpatient stay in participating geriatric or psychogeriatric departments. Clinical staff will screen patients for eligibility using a standardized screening form (checklist). If inclusion criteria are met and the patient expresses interest, the responsible BRIDGE team will be notified and will contact the patient and provide detailed study information. Patients will then be enrolled into BRIDGE within a time frame of four to ten days prior to discharge. All study participants will provide written informed consent prior to study participation and will subsequently be randomized to the IG (which receives the BRIDGE intervention) or the CG (which receives usual care).

### Inclusion and exclusion criteria

Persons who meet the following inclusion criteria will be eligible for study participation: age ≥ 65 years, inpatient or partial inpatient stay in one of the participating clinical study centers, community-dwelling, Geriatric Depression Scale (GDS-15; [33]) sum score ≥ 4, Mini-Mental State Examination (MMSE; [34]) sum score ≥ 20, multimorbidity (≥ 3 chronic mental or physical disorders), care level < 5 according to the German health system (with levels ranging from 1 = low need for help to 5 = most severe need for help), indication for behavioral and physical activation determined by the treating physician with respect to person-intervention fit (e.g., motivation and openness to change), statutory health insurance, mental capacity to consent. Participants will be excluded from participation if they are suffering from a terminal disease.

### Selection bias

To estimate potential selectivity in participant inclusion, reasons for non-participation and sociodemographic variables will be obtained from patients who decline study participation. These data will be collected through voluntary self-report using a paper-and-pencil questionnaire. In addition, interviews will be conducted with the clinical staff to capture participants’ reasons for declining participation. These data will be collected progressively over the course of eight months with each of the seven study centers collecting data for a two-month period. The two-month periods are randomly allocated to the study centers. Based on the data, a selectivity analysis will be conducted to identify potential confounders by comparing participants and non-participants. Any potentially identified confounders will be adjusted for in the main analyses. To account for potential selective dropout, an (ITT) approach has been chosen, i.e., all participants with available endpoint data from at least one measurement point will be included in the analyses.

### Clinical monitoring

The study will be accompanied by clinical monitoring to ensure adherence to the protocol and the quality of data collection. Hence, the Clinical Trial Center Würzburg ( = Zentrale für Klinische Studien am Universitätsklinikum Würzburg) will perform periodic monitoring visits at the participating sites following a monitoring plan with three visits per study center.

### Randomization and blinding

A stratified block randomization at the individual level with variable block length will be conducted for assignment to the IG or CG by the independent evaluator (IGES Institute, Berlin, Germany). Allocation will follow a 2:1 ratio, allowing for subgroup comparisons within the IG (basic vs. intensive version) without loss of statistical power. The study involves behavioral components — such as BA and PE — which by their very nature cannot be delivered without the participants and intervention facilitators being aware of the assigned condition. Hence, a blinded design is not feasible. Rigorous methodological strategies will be employed to ensure fidelity and reduce experimenter bias, such as objective outcome measures, standardized intervention protocols and no access to the random allocation sequence.

### Outcome measures

A comprehensive set of validated instruments will be employed to assess the primary and secondary outcomes across psychological, cognitive, physical, and functional health parameters. All primary and secondary outcomes are displayed in Table 1 together with their assessment time points over the course of the study.

**Table 1:**
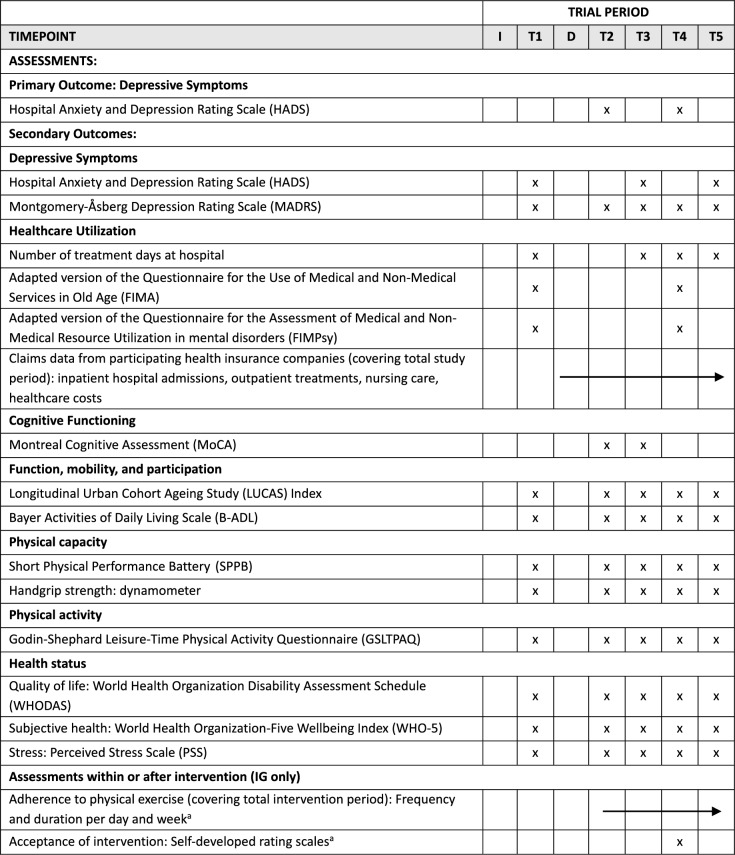
SPIRIT schedule of assessments

*I* Inclusion in the study, *D* Discharge from hospital, *T1* 4 – 10 days before discharge, *T2* ~ 7 days after discharge, *T3* 3 months after discharge, *T4* 6 months after discharge, *T5* 9 months after discharge

^a^Part of process evaluation

### Primary outcome

Maintenance or improvement of (partial) inpatient treatment results regarding depressive symptoms from immediately after discharge (approximately 7 days after discharge) (T2) to six months post-discharge (T4), assessed using the Hospital Anxiety and Depression Rating Scale (HADS; [35]).

### Secondary outcomes

Reduction of depressive symptoms (T1 – T5), reduction of healthcare utilization, increased/maintained cognitive performance, increased/maintained physical function, mobility, and participation, increased/maintained physical capacity, activity, and health (quality of life, emotional well-being, reduced perceived stress).

Depressive symptoms will be assessed using the HADS (self-rating) complemented by the Montgomery-Åsberg Depression Rating Scale (MADRS; [36]) to include clinician rating. Health-related medical and non-medical service utilization will be evaluated by the number of treatment days at hospital along adapted versions of the Questionnaire for the Use of Medical and Non-medical Services in Old Age (FIMA; [37]) and the Questionnaire for the Assessment of Medical and Non-medical Resource Utilization in Mental Disorders (FIMPsy; [37]) complemented by claims data from participating health insurance companies. Furthermore, cognitive functioning will be measured using the Montreal Cognitive Assessment (MoCA; [38]), a screening tool for mild cognitive impairment. Functional health, mobility, and participation will be assessed using the Longitudinal Urban Cohort Ageing Study (LUCAS) Index [39]. Ability to perform activities of daily living (ADLs) will be assessed using the Bayer Activities of Daily Living Scale (B-ADL; [40]). Concerning physical capacity the Short Physical Performance Battery (SPPB; [41]) will be used to assess lower extremity functioning. Hand grip strength [42] will be measured using a hydraulic hand dynamometer (Jamar) as an indicator of overall muscle strength. The Godin-Shephard Leisure-Time Physical Activity Questionnaire (GSLTPAQ; [43]) will be used as a self-report measure of physical activity. Overall health status will be assessed using the World Health Organization Disability Assessment Schedule (WHODAS; [44]), and the World Health Organization-Five Well-Being Index (WHO-5; [45]) as measures of quality of life complemented by the Perceived Stress Scale (PSS-10; [46]).

Data will be collected at five time points in order to assess short- and medium-term effects of the BRIDGE intervention. The time points will be identical for the IG and CG, and are as follows (T1): 4–10 days before discharge from inpatient stay; (T2): ~ 7 days after discharge (start of the intervention in the home); (T3): 3 months after discharge (at the end of the intervention); (T4): 6 months after discharge (at the end of the check-up calls in the intensive version); and (T5): 9 months after discharge (end of follow-up). Methods include self-reports, structured interviews, and validated assessment tools. Assessments will be conducted in inpatient and outpatient settings either through self-report or by the members of the BRIDGE team. Data collection by the BRIDGE team will take place according to a standardized protocol and documented in an electronic case report form (eCRF) using the web-based application REDCap (https://project-redcap.org/). Self-report data collected via self-administered paper-and-pencil questionnaires will be sent directly to the independent external evaluation institute (IGES Institute, Berlin, Germany). All data will be pseudonymized for data analysis.

### Intervention

**Fig. 3:**
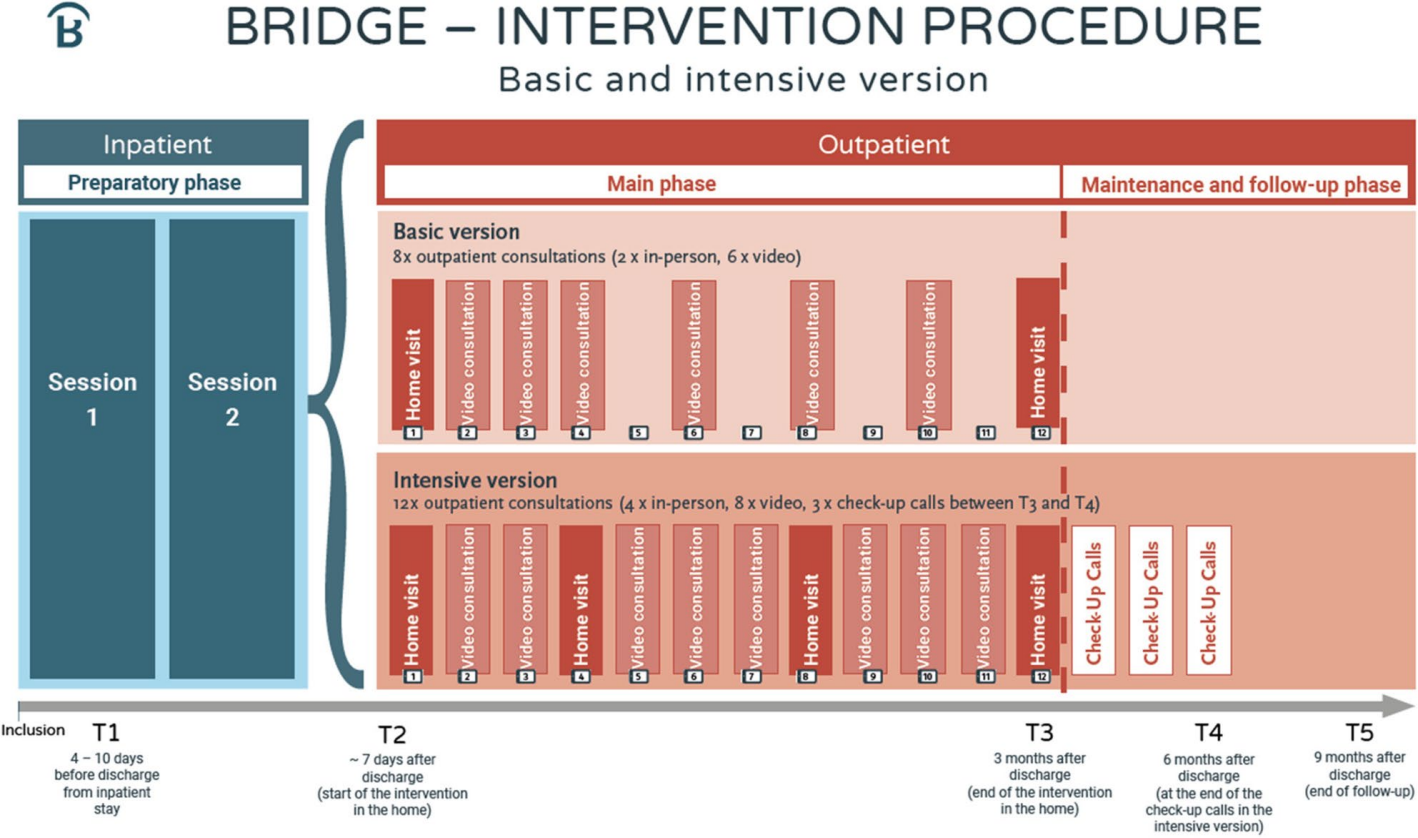
Overview of the BRIDGE intervention including an inpatient preparatory phase, an outpatient main phase followed by a maintenance and follow-up phase. The intervention is prepared during the two inpatient sessions and participants are allocated to either the basic or intensive version based on the severity of depressive symptoms (MADRS score ≥ 20 for the intensive version). Both versions consist of blended care comprising home visits, video consultations and home-based guided PE videos and differ only in the number of consultations (basic = 8 x obligatory consultations: 2 x home visits and 6 x video consultations, 12 guided PE videos; intensive = 12 x obligatory consultations: 4 x home visits and 8 x video consultations, 12 guided PE videos). The intensive version includes three additional monthly check-up calls following the intervention

**Fig. 4:**
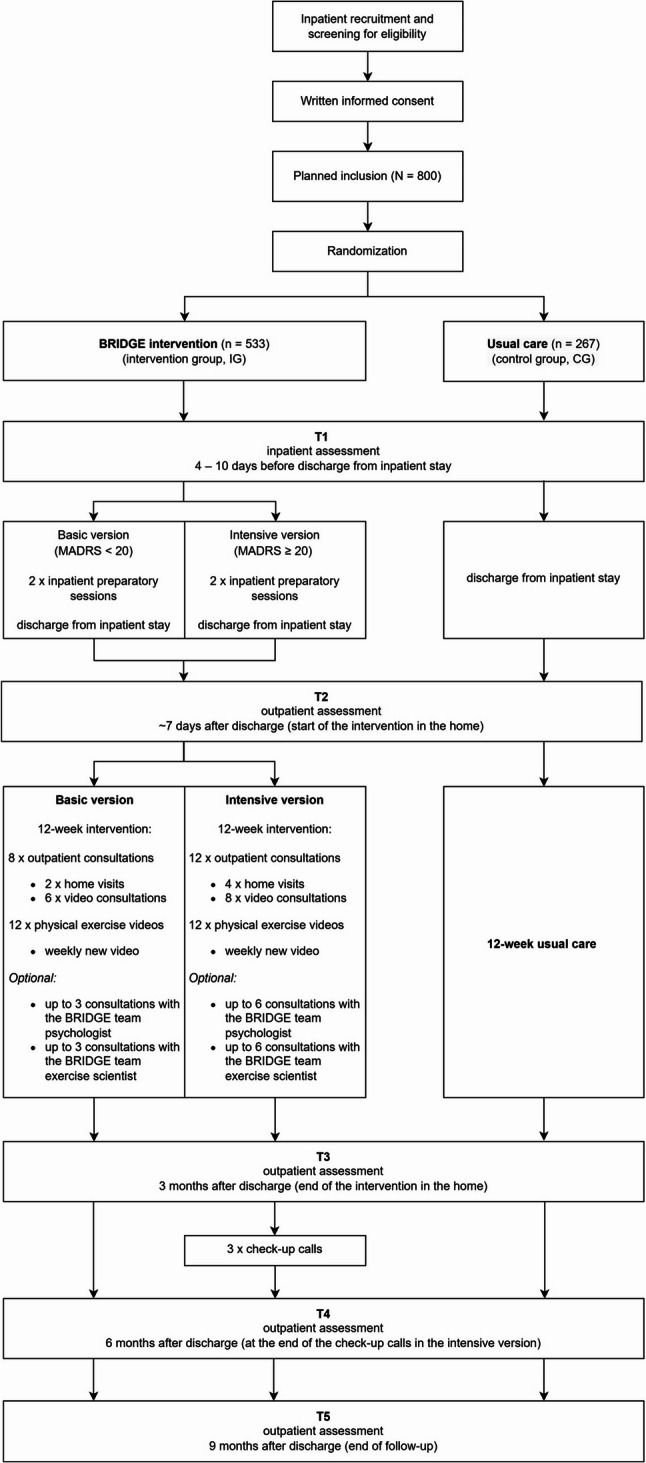
BRIDGE study flow chart

The BRIDGE intervention is a structured, manualized, interdisciplinary treatment program designed to support older, multimorbid participants with depressive symptoms during the critical transition from inpatient to outpatient care. It is embedded in an interdisciplinary and multiprofessional approach to optimally combine knowledge on psychological and exercise-based interventions for older adults with depressive symptoms. A distinctive feature of the BRIDGE intervention are the two interconnected and interacting core components, which comprise elements of behavioral activation and guided home-based physical exercise and are specifically developed and tailored for this vulnerable population. The BRIDGE blended care intervention spans 12 weeks per participant and is delivered using a modular, manualized treatment protocol, both in-person (e.g., home visits) and through a digital e-health solution (e.g., video consultation, PE videos, and mood monitoring) supported by a specifically developed workbook (BRIDGE workbook). Based on the severity of depressive symptoms, as assessed using the MADRS at T1 assessment, participants allocated to the IG will be assigned to either (i) a basic version (MADRS score < 20) or (ii) an intensive version (MADRS score ≥ 20). The two intervention versions are generally similar in content but comprise a different frequency of home visits and video consultations (Fig. 3 & 4).

i. **Basic** = 8 x obligatory consultations: 2 x home visits and 6 x video consultations, 12 guided PE videos;
ii. **Intensive** = 12 x obligatory consultations: 4 x home visits and 8 x video consultations, 12 guided PE videos + 3 monthly check-up calls following the intervention period.

Both versions are accompanied by two inpatient modules that prepare patients for the transition, introduce the rationale for BRIDGE, and familiarise patients with the electronic device enabling participation in video consultations without the need for technical expertise. All participants in the intervention group will receive the so-called BRIDGE device, a 11,0’’ tablet operated via a docking station (enna systems GmbH, Munich, Germany). This device is connected to the internet and enables the video consultations, delivers the PE videos that participants will be instructed to follow at home, and mood monitoring two times per day. Participants will receive a set of cards (credit card format with NFC technology) which, when placed on the docking station, activate video consultations with the BRIDGE team or one of 12 physical exercise videos. This solution allows for digital participation without technical expertise, as the functions of the cards are depicted on the cards themselves, meaning that neither touch screen use nor online competencies are needed to start the videos consultations and exercise videos. Additionally, participants will receive a BRIDGE workbook. 

### Behavioral activation component of the BRIDGE intervention

The BRIDGE intervention incorporates a structured BA framework, specifically adapted to the psychosocial and functional needs of older, multimorbid individuals with depressive symptoms. The BA component emphasizes the systematic identification of and engagement in rewarding activities to counteract depressive symptomatology in line with the behavioral theory of depression [47] by use of specific Behavioral Change Techniques (BCT). BCT´s are following the BCT taxonomy v1 [48] comprising Goal setting (behavior) [1.1] (e.g., agree on engaging in three daily positive activities and reach agreement about the goal), and Action planning [1.4] (e.g., written scheduling of pleasant activities including physical exercise videos using the BRIDGE workbook). Self-monitoring of behavior [2.3] (e.g., by ticking off in the BRIDGE workbook if the planned activities have been performed), and Self-monitoring of outcome(s) of behavior [2.4] (e.g., daily rating of the current mood state on a 7-point visual analogue scale (VAS) using facial expressions ranging from “very unhappy" to "very happy” in the morning and evening automatically provided by the BRIDGE device; rating of mood changes due to a performed activity on a 7-point VAS using facial expressions ranging from “very unhappy" to "very happy” in the BRIDGE workbook), along Feedback on outcome(s) of behavior [2.7] and Social reward [10.4] (e.g., monitoring of pleasant activities including physical exercise videos, mood monitoring and discussion of changes with the BRIDGE team). Moreover, Social support practical [3.2] and emotional [3.3] (e.g., during home visits and video consultations), Information about emotional consequences [5.6] (e.g., effect of physical exercise on mood), Information about health consequences [5.1] (e.g., effect of physical exercise on physiological fitness) provided by the BRIDGE team. Additionally, Instruction on how to perform the behavior [4.1] (e.g., written and verbal instructions provided in the BRIDGE workbook and via the BRIDGE team), Prompts/cues [7.1], Habit formation [8.3], and Self-reward [10.9] in form of the Tiny Habits^®^ method [49]). The BCT´s are completed by Self-talk [15.4] (e.g., self-formulated positive sentences written into the BRIDGE workbook after completing an activity or coping with barriers) and Verbal persuasion about capability [15.1] by the BRIDGE team during home visits and video-consultations (e.g., telling the participants that they can successfully perform the desired behavior, arguing against self-doubts and emphasizing their abilities and progress) and overall Reduce negative emotions [11.2] (e.g., advising participants to use BA components and PE videos to enhance or stabilize their mood) 

In addition to the use of BCTs, one prominent feature of BA in the BRIDGE intervention is its integration of the Tiny Habits^®^ method [49], which is based on evidence from the Positive Experience Program (PEP) for Depressed Older Adults [50]. While BA emphasizes the value of increasing engagement in everyday positive activities to lift mood, the Tiny Habits^®^ method is a straightforward, evidence-based framework for integrating new behaviors into existing routines. It employs a structured approach called Tiny Habits Recipes, which follow the format: “*After [Anchor Moment]*,* I will [Tiny Behavior].*” The Anchor Moment refers to an action that is already reliably part of one’s daily routine, serving as a cue for the new behavior. The Tiny Behavior is the desired action, scaled down to require minimal time and effort. For example, a recipe might be: “*After I brush my teeth in the morning*,* I will do five shoulder rolls to mobilize my shoulders*”. To reinforce the new behavior, a celebration is performed immediately afterward, such as thinking or saying out loud, “*It feels great to be mobilized!*” or patting oneself on the shoulder, to evoke positive emotions and a sense of success. This reinforcement strengthens the connection between the Anchor Moment and the Tiny Behavior, promoting automation of BA. The latter part reinforces the immediate awareness of the synergies between activity and mood. Additionally, the method involves a focus on behaviors that individuals genuinely want to adopt rather than those they feel they should or must do. Research supports the effectiveness of the Tiny Habits^®^ method (e.g [51]). It utilizes implementation intentions [52], which have been shown to enhance goal achievement by increasing the mental accessibility of cues (Anchor Moments) and solidifying the link between cues and planned actions (Positive Tiny Behaviors). Moreover, small, simple, and intrinsically enjoyable behaviors are expected to be more consistently performed and more easily formed into habits. The behavioral activation methods of the BRIDGE program are especially well-suited for multimorbid older adults experiencing subsyndromal or clinical depression, for whom low burden and positive reinforcement strategies are critical to maintaining engagement and promoting emotional well-being.

The inclusion of guided, video-based PE further strengthens the activation framework of the BRIDGE program and is supported by educational materials that promote safe and accessible home-based exercise. These strategies are intended to help participants to maintain behavioral and physical activation even when motivation or mood decline.

### Physical exercise component of the BRIDGE intervention

As a core pillar of the BRIDGE intervention, physical activity is promoted through the systematic implementation of daily video-guided exercise sessions. The exercise sessions will be performed independently by participants at home using the BRIDGE device (tablet device) to play the videos. The videos were developed specifically for the BRIDGE intervention based on the expertise of exercise scientists and psychologists, oriented towards established physical exercise programs for older adults such as the Otago Exercise Program [53, 54], VIVIFRAIL [55], PROMOTE [56, 57], and PROGRESS [58]. They are designed to support both physical and mental health and are tailored specifically for older, multimorbid adults experiencing depressive symptoms and are supplemented by mind-body exercises (e.g., yoga exercises, progressive muscle relaxation, and breathing meditation). Exercises are designed to be effective in reducing depressive symptoms [59] also in this population [17, 60–62]. Participants will receive a total of 12 guided PE videos, with one new video per intervention week. The PE videos are available in a standing and sitting variant, depending on individual physical abilities (e.g., balance, walking), as assessed using the SPPB [41] at T1 assessment. Participants with an SPPB score below the cut-off of 7 will be assigned to the sitting PE videos. Each exercise video lasts approximately 20 min and follows a standardized structure, consisting of a welcome and warm-up phase, a main training phase, and a cool-down phase with a relaxation focus. The PE in the videos are verbally instructed (BCT: Instruction on how to perform the behavior [4.1]) and visually demonstrated (BCT: Demonstration of the behavior [6.1]) by a middle-aged female instructor, serving both as a facilitator and a role model (BCT: Behavioral practice and rehearsal [8.1], e.g., prompt participants to include physical exercises in their daily life; [12.6] Body changes, e.g., prompt aerobic, strength, and coordination exercises, and relaxation training). In more detail, the exercise videos are grounded on a multicomponent training approach that integrates whole-body strength training, endurance exercises, flexibility, coordination activities, dual-task exercises (e.g., simultaneous motor-cognitive tasks), and elements of mind-body exercises. In the latter, a distinct emphasis is placed on the mind-body connection, which is operationalized through integrated elements of relaxation and mindfulness (e.g., breathing meditation, progressive muscle relaxation). Mind-body exercises typically include inward-focused attention on breathing and body awareness (proprioception), which may facilitate antidepressant effects [60]. Accumulating evidence suggests that mind-body exercise approaches exert the most robust antidepressant effects in older adults [16, 63]. The overall exercise content is intentionally progressive: Over the 12-week course of the intervention, each new video incrementally increases the physical and cognitive demands, either through the introduction of novel exercises or the modification and intensification of previous ones. This structured progression is aimed to reduce depressive symptoms and enhance physical fitness, which seems to be more effective with more intense exercise [16, 64], and to promote neuroplasticity through ongoing cognitive-motor stimulation [65]. To support motivation, adherence, and emotional engagement, all videos are narrated by the same female instructor, who delivers scripted personalized and encouraging messages, reinforcing participants’ self-efficacy and mindfulness and promoting a self-empowering narrative throughout the exercise videos. Taken together, the structure, content, and scripted instructions facilitate safe participation while providing a familiar and predictable format that supports adherence over time. Although participants engage with the exercise videos independently, their experience is closely monitored and reflected upon during home visits and video consultations, during which members of the BRIDGE team review adherence and feedback related to the video exercises (assessed via the enna.care app) and identify and tackle barriers to participation, for example by adjusting the physical activity component as needed based on individual abilities (e.g., an exercise part cannot be performed due to a physical impairment or pain), safety considerations, or changes in physical health.

This dual approach — self-guided yet therapeutically supported — encourages autonomy while maintaining clinical monitoring, thereby maximizing engagement and potential treatment effects.

### Intervention delivery: the BRIDGE team

An essential component of the BRIDGE intervention is the establishment of an interdisciplinary and multiprofessional BRIDGE team at each study center. The BRIDGE team consists of a nurse, a psychologist, and a exercise scientist. This interprofessional structure will be maintained throughout the intervention period to ensure the optimal and continued integration of nursing, psychological, and physical activity expertise in addressing the complex needs of older adults with depressive symptoms and multimorbidity.

Prior to implementation, all BRIDGE team members will complete a standardized, role-specific, and interprofessional training program, which includes pre-recorded instructional videos and interactive online sessions based on a structured intervention manual. The training will ensure consistent delivery of the intervention across study sites and professional roles.

The BRIDGE nurse will serve as the primary contact person for participants and as the main intervention provider. Responsibilities of this role include home visits, digital video consultations, facilitation of an activity schedule, encouragement of mood monitoring, implementation of clinical assessments (T1 – T5), and documentation of intervention components as well as clinical observations. The nurse will establish a relationship of trust and cooperation with participants, characterized by empathy and individually tailored support. This includes monitoring depressive symptoms, promoting BA, and facilitating access to further services (e.g., exercise groups, community centers). In acute situations (e.g., psychological crises or serious physical complaints), the nurse will coordinate with team members to initiate appropriate emergency procedures. The exercise scientist will support and supervise the nurse in the assessment of physical functioning (e.g. handgrip strength, SPPB), advise on individual tailoring of the exercise content across the intervention, and educate participants on the health benefits of PE. They may also offer direct patient consultation if needed (see Fig. 4) and contribute to decision-making during physical health emergencies in accordance with their professional scope. The psychologist will ensure both quality and fidelity of the BA. They will support the nurse in the assessment of psychological symptoms and cognitive and global functioning while also supervising the nurse in terms of adapting and individually tailoring the BA to fit specific patient needs. Additionally, the psychologist will provide psychoeducational information on depression and the principles of BA for the participants. The psychologist may also provide participants with direct consultations if needed (see Fig. 4). In the case of a mental health crisis, the psychologist will be responsible for an initial risk assessment and will work with the team and healthcare providers to coordinate an appropriate and timely response in accordance with German emergency medical regulations.

#### Interprofessional collaboration

Interprofessional collaboration within the BRIDGE team is an integral part of the BRIDGE intervention. All team members will complete joint training and engage in structured interprofessional meetings during the intervention period. These weekly meetings will enable shared decision-making, review of clinical findings, and discussion of implementation barriers (e.g., reduced motivation, comorbid physical conditions). Each team member will contribute equally to these discussions, fostering collaborative learning and integrated care delivery. Additionally, each BRIDGE team will participate in monthly online supervisions, provided in an individual setting (focusing on one BRIDGE team) and a group setting (including all BRIDGE teams across study centers), led by two BRIDGE project team members (an exercise scientist and a psychologist).

### Control group (CG)

Participants allocated to the CG will receive usual care consisting of health and care services routinely available to older adults in the German healthcare system. Following the final assessment (T5, nine months post-discharge), participants will be compensated with a €30 voucher redeemable for a large variety of goods online (wunschgutschein.de), whereas participants in the IG will not receive monetary compensation. 

### Outcome evaluation

Five assessment time points (T1 – T5) are planned to assess the short- and medium-term effects of the BRIDGE intervention. These time points are identical for the IG and the CG. Table 1 provides an overview of the outcome evaluation endpoints and their operationalization, including the corresponding data sources. For most endpoints, the post-discharge time point T2 (start of the intervention in the home) is used as the baseline, since the (partial) inpatient setting at T1 could bias the baseline assessment. The primary endpoint is defined as the maintenance or improvement of (partial) inpatient treatment outcomes regarding depressive symptoms from post-discharge (T2) to six months post-discharge T4 (at the end of the check-up calls in the intensive version). Depressive symptoms will be assessed at all time points (T1 through T5) via both self-report (e.g., HADS) and clinician rating (e.g., MADRS) to additionally evaluate the secondary endpoint of depressive symptom reduction over the full follow-up period. Physical functioning, physical activity, quality of life, perceived stress, and emotional well-being will also be assessed at all five time points (T1 to T5). To prevent training effects, cognitive functioning will be measured at T2 and T3 (end of the intervention in the home) only; no further assessments of cognitive functioning are planned. The number of days of (partial) inpatient days after the initial discharge will be documented by the BRIDGE team at T3, T4, and T5.

### Process evaluation

The process evaluation includes surveys and interviews with participants as well as an online survey and focus groups with the BRIDGE team members as intervention providers. Both primary data (from participants and BRIDGE teams) and secondary data (eCRF and usage of BRIDGE device) will be analyzed.

### Evaluation by participants

Acceptance and satisfaction with the BRIDGE intervention will be assessed at the end of the intervention through self-report questionnaires at T3 and T4. The fidelity of the BRIDGE intervention will be documented via the eCRF, supplemented by usage data recorded by the BRIDGE device (e.g., frequency and duration of physical exercise videos used). Custom-developed questionnaires, informed by established patient satisfaction instruments (e.g. [66, 67]), will be used to evaluate specific components of the intervention such as the blended care approach (mix of home visits and video consultations), physical exercise, workbook use, and the digital components.

Additionally, an in-depth process evaluation will be conducted, exploring the subjective change processes perceived by participants in their daily lives as a result of their participation. In the first step, a subset of participants from the intervention group will be invited to take part in structured interviews after completing the intervention (after T3) to explore their subjective experiences. To achieve theoretical saturation, approximately 20 participants are expected to be required. For pragmatic reasons (see below), recruitment will focus on those who complete the intervention the earliest, ensuring variation in gender, hospital site, intervention format (basic vs. intensive version), and degree of physical impairment. The interviews will be audio-recorded and fully transcribed. Using qualitative content analysis [68], categories will be developed inductively from the textual material. These categories will represent different types of subjective change processes (e.g., “rediscovering resources,” “defining life goals”). Based on the categories, an instrument will be developed to conduct a short, problem-centered interview, which will then be used with all IG participants who did not take part in the initial 20 interviews. This interview will be administered at the 3-month follow-up (T4; six months post-discharge and the end of the check-up calls in the intensive version).

### Evaluation by BRIDGE teams

An online survey of all BRIDGE team members will be conducted during the final quarter of their study involvement (or upon exit, but no earlier than six months after the project’s start). The survey will assess acceptance and perceived utility of the care model for routine practice. Additionally, two focus groups will be held to explore interdisciplinary and cross-sector collaboration as well as the potential to integrate the BRIDGE intervention into existing care structures in Germany. Each group will include BRIDGE team members of all sites (~ 21 participants in total), ensuring representation across regions (urban and rural). Topics will be derived from project experience and may include urban–rural disparities in care infrastructure, the impact of initial treatment setting (e.g., geriatrics vs. psychogeriatrics), and differences between partial and full inpatient groups.

### Health economic evaluation

The health economic evaluation aims to assess the cost-effectiveness of the BRIDGE intervention compared to usual care from a healthcare system perspective. A cost-effectiveness analysis (CEA) will be conducted using the incremental cost-effectiveness ratio (ICER), which relates the difference in total costs between the IG and the CG to the difference in observed benefits. Effectiveness will be derived from the primary outcome and selected secondary outcomes.

$$\:ICER=\frac{IG\:costs-CG\:costs}{\mathrm{I}\mathrm{G}\:\mathrm{b}\mathrm{e}\mathrm{n}\mathrm{e}\mathrm{f}\mathrm{i}\mathrm{t}-\mathrm{C}\mathrm{G}\:\mathrm{b}\mathrm{e}\mathrm{n}\mathrm{e}\mathrm{f}\mathrm{i}\mathrm{t}}$$

## Operationalization of costs

Costs are primarily operationalized via healthcare and long-term care utilization. Primary data on healthcare and long-term care utilization will be collected through structured interviews conducted by the BRIDGE team six months post-discharge (T4). The interview follows standardized instruments adapted from validated tools such as the FIMA and FIMPsy, covering the following categories: outpatient medical care (e.g. physician visits), inpatient care (e.g. hospital days), (psychiatric) outpatient services of hospitals (e.g. long-term care services), therapeutic services (e.g. physiotherapy), medical devices (e.g. wheelchairs), pharmaceuticals (limited to psychotropic medications).

To validate and adjust for potential self-reporting bias, anonymized routine data will be obtained from participating statutory health insurance providers for approximately 15% of participants. The data will cover costs and health care as well as long-term care utilization in key healthcare sectors across two quarters before the enrollment quarter, the enrollment quarter, and the two quarters after the enrollment quarter (in total seven quarters).

Costs of care utilization will be determined using a combination of standard unit costs derived from publicly available national databases (e.g. Federal Statistical Office), routine claims data from health insurance providers, and internal cost data from the BRIDGE consortium for estimating the intervention’s implementation costs per patient. All costs will be adjusted to the year of analysis using inflation indices or healthcare-specific adjustment factors, as appropriate. The cost structure will include:

- Intervention group (IG):
  - K-11: Costs of the new BRIDGE intervention
  - K-12: Costs of usual care
  - Total costs = K-1 = K-11 + K-12
- Control group (CG):
  - K-21: Costs of usual care
  - Total costs = K-2 = K-21

### Operationalization of benefits

Benefits of BRIDGE (IG) and standard care (CG) are derived from the outcome evaluation. Three different benefits are estimated and analyzed separately:

- N-1: achievement of the primary endpoint
- N-2: index of achievement of selected secondary endpoints (sum-score); reduction of depressive symptoms, increase or maintenance of physical activity, increase or maintenance of physical functioning, increase or maintenance of quality of life, increase or maintenance of cognitive performance, reduction of perceived stress, increase or maintenance of emotional well-being
- N-3: number of days in inpatient or day clinic stays

### Planned statistical analyses

Descriptive statistics will first be calculated for all outcome measures. To test the predefined hypotheses regression models — such as mixed models for repeated measures (MMRM) — will be employed, using appropriate link functions depending on the distribution of the outcome variables.

These regression models allow for flexible modelling of outcomes, accounting for potential confounding variables at both the individual and hospital levels — particularly in cases where randomization does not result in balanced groups or where selectivity analyses reveal relevant differences. Additionally, using a multilevel modelling approach enables to account for the clustering of participants within study centers.

The analytic strategy will follow an intention-to-treat (ITT) approach, enabling inclusion of participants despite missing data in the longitudinal follow-up and avoiding case-wise deletion due to attrition. Missing data will be handled using appropriate imputation or model-based estimation techniques to ensure robustness of the statistical analyses.

Subgroup analyses will be conducted for all primary and secondary endpoints to examine differences in intervention effects between the basic and intensive version of the BRIDGE intervention. This will involve estimating the interaction effect using a three-level grouping variable (IG-basic, IG-intensive, CG) to help identify the most appropriate target population for the intervention. Exploratory analyses will examine dose–response and adherence effects using data from the BRIDGE devices and app, which track the frequency and duration of exercise video use and video consultations. All statistical analyses will be conducted using the open-source software R (https://www.r-project.org/).

## Discussion

### Summary

The BRIDGE intervention is a structured, manualized, interdisciplinary treatment program designed to support older, multimorbid patients with depressive symptoms during the critical transition from (partial) inpatient to outpatient care. The intervention combines two evidence-based therapeutic strategies — behavioral activation (BA) and physical exercise (PE) — in a three-month multimodal program designed to maintain or improve treatment outcomes regarding depressive symptoms. The intervention will take place partly in person and partly via video consultations in addition to a continuous online physical exercise program. The primary objectives of the BRIDGE trial are to assess the efficacy, cost-effectiveness, and feasibility of this combined intervention in reducing depressive symptomatology and promoting functional engagement in everyday life.

#### Synergistic combination of behavioral activation & physical exercise

A core assumption underlying the intervention is that systematic activity planning and engagement — as derived from BA — can disrupt maladaptive patterns of avoidance and inactivity that are characteristic of depressive states [69, 70]. Simultaneously, the PE component is expected to produce both direct mood-enhancing effects and indirect benefits via improvements in physical health, with superior effects for the combination of BA and PE compared to one method alone [28]. The primary assumption is that the BRIDGE intervention will improve, or at least maintain, the reduction in depressive symptom severity (achieved during (partial) inpatient care) in this particularly vulnerable population, which typically faces barriers in accessing mental healthcare.

Moreover, the intervention focuses on a vulnerable context of the healthcare setting – the transition from inpatient care to home-based living – with the aim to promote sustained improvements across multiple domains of health and everyday functioning in older multimorbid adults. One goal of the intervention is to reduce the number of treatment days spent in hospital settings, thereby minimizing the need for recurrent inpatient admissions in the long-term. In addition, the program seeks to increase or maintain levels of physical activity by encouraging regular, structured exercise as part of daily routines. Physical capacity is another key target, with a particular focus on enhancing or preserving lower body strength, balance, and handgrip strength as indicators of overall physical fitness and resilience. Furthermore, the intervention is designed to support the maintenance or improvement of everyday functioning, mobility, and participation in social and community life. These outcomes are assessed by examining the ability to perform instrumental and basic activities of daily living and by evaluating levels of independence and engagement in routine tasks. Cognitive performance is also addressed, with the aim of stabilizing or enhancing memory, attention, and executive functioning over time. Alongside these physical and cognitive outcomes, the intervention emphasizes improvements in overall quality of life, including domains such as autonomy, interpersonal relationships, and the capacity to carry out meaningful roles. Emotional well-being is a further focus, with efforts to increase positive mood and life satisfaction while simultaneously reducing symptoms of psychological stress. Taken together, these multidimensional outcomes reflect the comprehensive scope of the BRIDGE intervention and its potential to support holistic recovery in later life.

#### Multidisciplinary team approach

The multidisciplinary structure of the BRIDGE teams represents a core principle of the intervention, enabling comprehensive, patient-centered care for older adults with multimorbidity and depressive symptoms. By integrating expertise from nursing, psychology, and exercise science, the intervention addresses key biopsychosocial domains known to influence mental health outcomes in later life. The continuity provided by a fixed interprofessional team at each study center promotes consistency in intervention delivery, while structured interprofessional collaboration — through joint training, weekly case conferences, and supervision — enhances communication and coordination across disciplines. This model supports tailored clinical decision-making, improves intervention fidelity, and facilitates shared problem-solving in complex cases. Importantly, placing the nurse at the center of intervention delivery ensures regular, trust-based contact with participants, while allowing specialist roles (psychologist and exercise scientist) to contribute targeted expertise. This stepped and cooperative care approach reflects current recommendations for collaborative care in geriatric mental health and may support scalability and transferability of the BRIDGE intervention to routine settings. By operationalizing a multidisciplinary intervention team supported by structured training and supervision, BRIDGE is positioned to meet the complex needs of its target population in a feasible and sustainable manner.

#### Digital intervention components

The integration of digital components within the BRIDGE intervention represents a key innovation. Using a simplified tablet-based e-health platform (BRIDGE device), participants can engage with key elements of the intervention — including video consultations, mood monitoring, and structured PE sessions — outside of home visits. The use of tactile, card-based hardware (enna system) supports ease of use, especially for participants with limited digital literacy. By delivering core intervention modules both in-person during the home visits and digitally via the BRIDGE device over the 12-week intervention period, BRIDGE promotes flexible, blended care that can be adapted to individual needs and logistical constraints. This approach not only supports adherence and self-management but may also enhance scalability and reach in future routine care implementations.

#### Feasibility and implementation potential

In addition to evaluating clinical outcomes, the study includes process evaluations to assess key indicators of feasibility, acceptability, and fidelity under routine care conditions. This includes participant adherence, satisfaction with intervention materials (e.g., BRIDGE workbook, Tiny Habits^®^ method and guided home-based exercise videos), and perceived burden. The process evaluation also captures perspectives from both participants and healthcare providers through surveys, interviews, and focus groups. These insights will be critical in identifying facilitators and barriers to implementation, guiding potential adaptation, and informing the scalability of BRIDGE in broader clinical settings.

Moreover, a health economic evaluation will assess the cost-effectiveness of the BRIDGE intervention relative to usual care. By combining primary data on healthcare resource use with routine data from statutory health insurance providers, the study will generate a comprehensive estimate of direct medical costs. The incremental cost-effectiveness ratio (ICER) will relate these costs to benefits operationalized via clinically relevant outcome measures, including maintenance or improvement of depressive symptom reduction and quality of life. These analyses will provide valuable evidence regarding the economic viability of BRIDGE and its potential for adoption into standard care pathways.

#### Limitations

Despite the promising effects of BA and PE for counteracting depressive symptoms, and the practical relevance of BRIDGE, several potential limitations must be considered. First, due to the nature of an RCT, the CG receiving usual care may be biased towards higher dropout rates, thus limiting comparability between the IG and the CG.

Second, because it is not feasible for healthcare provision and data collection to be conducted by two separate persons, detection bias cannot be mitigated through this approach. Distortion in data collection due to positive expectations regarding the IG is therefore possible for data collected through interviews or observer ratings. Concurrently, spillover effects that could counteract potential detection bias may be anticipated in the CG due to the shared clinical context. Further, repeated personal data collection in interview format by the nursing professionals may lead to measurement-induced reactivity.

Regarding generalizability, the BRIDGE intervention is tailored to older multimorbid adults with depressive symptoms transitioning from inpatient settings to their homes. While this represents a clinically relevant and underserved population, the findings may not generalize to younger cohorts or to older adults without recent hospitalization. Cultural and linguistic factors may also influence the acceptability of certain components (e.g., the style of PE videos or the nature of BA techniques), suggesting the need for cultural adaptation in future studies.

## Conclusion

The BRIDGE intervention represents a novel, innovative, evidence-informed intervention aimed at enhancing mental health and functional outcomes in older multimorbid adults with depressive symptoms during the sensitive transitional period from inpatient to outpatient care. By integrating daily behavioral and physical activation strategies in terms of regular positive routines and PE, the program bridges clinical care with a meaningful home-based, low-threshold, and digitally supported activation program oriented to everyday life. In summary, the BRIDGE study evaluates a complex intervention in older adults by embedding comprehensive outcome and cost-effectiveness analyses. This RCT has the potential to generate actionable insights into evidence-informed healthcare planning for real-world settings and policy development, informing large-scale implementations in diverse care contexts in the future.

## Supplementary Information

Supplementary Material 1.

## Data Availability

No datasets were generated or analysed during the current study.
